# Predicting distribution of *Zanthoxylum bungeanum* Maxim. in China

**DOI:** 10.1186/s12898-020-00314-6

**Published:** 2020-08-11

**Authors:** Zhihang Zhuo, Danping Xu, Biao Pu, Rulin Wang, Meng Ye

**Affiliations:** 1 College of Life Science, China West Normal University, 1 Shida Road, Nanchong, 637002 China; 2 College of Food Science, Sichuan Agricultural University, 46 Xinkang Road, Yaan, 625014 China; 3 College of Forestry, Hainan University, 58 Renmin Avenue, Haikou, 570228 China; 4 Sichuan Provincial Rural Economic Information Center, 6 Guanghua Village Street, Chengdu, 610072 China; 5 College of Forestry, Sichuan Agricultural University, 211 Huimin Road, Chengdu, 611130 China

**Keywords:** Environmental variables, MaxEnt model, *Zanthoxylum bungeanum* Maxim., Distribution area

## Abstract

**Background:**

With the growth of economic benefits brought by *Zanthoxylum bungeanum* Maxim. and the increasing market demand, this species has been widely introduced and cultivated in China. It is important to scientifically select suitable areas for artificial planting and promotion, and to understand the status and potential of *Z. bungeanum* resources.

**Results:**

The maximum entropy (MaxEnt) model and ArcGIS technologies were used to analyze the climatic suitability of *Z. bungeanum* based on known distribution data, combined with environmental data in China. *Z. bungeanum* was mainly distributed in subtropical and mid-eastern warm temperate regions. The total suitable area (high and medium suitability) accounted for 32% of China’s total land area, with high suitability areas composing larger percentage, reaching 1.93 × 10^6^ km^2^. The suitable range (and optimum value) of the key environmental variables affecting the distribution of *Z. bungeanum* were the maximum temperature in February of 2.8–17.7 °C (10.4 °C), the maximum temperature in March of 8.6–21.4 °C (16.3 °C), the maximum temperature in December of 2.5–17.1 °C (9.9 °C), the maximum temperature in November of 7.7–22.2 °C (14.5 °C) and the mean temperature in March of 3.2–16.2 °C (12.0 °C).

**Conclusions:**

The model developed by MaxEnt was applicable to explore the environmental suitability of *Z. bungeanum*.

## Background

*Zanthoxylum bungeanum* Maxim. is a small deciduous tree that belongs to the Rutaceae family. The fruit is purple-red and scattered with slightly raised oil spots. Its roots, stems, fruit and leaves can be used as raw materials for biomedicine, with antibacterial, anti-tumor, anti-inflammatory, analgesic and anti-oxidation effects [1–3]. The pericarp is famous for its pungent and numbing flavor, so it is also widely used as a seasoning. With the growth of economic benefits brought by *Z. bungeanum* and the increasing market demand, this species has been widely introduced and cultivated. In the process of introduction and cultivation, it is necessary to consider *Z. bungeanum*’s adaptability to local climatic conditions, to avoid the quality degradation and resource waste caused by inappropriate introduction. It is also important to scientifically select suitable areas for artificial planting and promotion, to understand the status and potential of *Z. bungeanum* resources.

Species distribution models use species distribution data and environmental data to estimate the distribution of a species based on a specific algorithm and to reflect the preference of a species to a habitat in the form of probability [4]. Although a variety of distribution models have been established, studies have shown that MaxEnt model is superior to other models in predicting with accuracy, especially in the case of incomplete species distribution data [5–7]. Maxent model is a niche model with good prediction effect. It demonstrates a strong capacity to distinguish the interaction of variables and cope with sampling deviation. It is simple and fast to operate and requires only a small sample size. The MaxEnt model has been applied in the simulation of pest and disease spread [8], the potential habitat quality estimation of endangered animals and plants [9], the risk assessment of invasive alien species [10], the prediction of suitable habitats for crop planting [11], the adaptation response to climate change [12], and good simulation results have been achieved. The MaxEnt model uses Jackknife to judge the importance of environmental factors and to quantitatively describe the effects of environmental factors on species habitats. However, there are few reports on the prediction of the suitable area of *Z. bungeanum* by MaxEnt in China which will restrict the future development of this species to a certain extent. We hypothesize that climate and topographical variables could be used to predict the suitable area of *Z. bungeanum*, and key environmental variables affecting the distribution would be obtained.

In this work, Maxent and ArcGIS technologies were used to analyze the environmental suitability of *Z. bungeanum* based on known distribution data, combined with environmental data in China. The key environmental variables affecting distribution and suitable growing areas were identified, which provided a scientific basis for practical introduction and cultivation of *Z. bungeanum* in the future.

## Results

### Dominant environmental factors

The contribution of each environmental factor to the suitable distribution area of *Z. bungeanum* was quantitatively calculated by the Jackknife test (Table 1). Variables with zero contribution were removed. Prec8 contributes the most to the distribution, reaching 21.3%. The other main contribution factors contributing more than 10% are tmax3 (20.3%), tmax11 (15.1%) and tmax2 (14.4%) with an accumulated percent contribution accounting for more than half of the total contribution (71.1%). The single factor contribution rate of all twelve main contribution factors is more than 0.3% with accumulated percent contribution reaching 99.9%.

**Table 1:** The contribution of each main contribution factor in MaxEnt modeling

Code	Environmental variables	Percent contribution/ %	Accumulated Percent contribution/ %
prec8	Precipitation of August	21.3	21.3
tmax3	Maximum Temperature of March	20.3	41.6
tmax11	Maximum Temperature of November	15.1	56.7
tmax2	Maximum Temperature of February	14.4	71.1
bio15	Precipitation Seasonality (Coefficient of Variation)	9.7	80.8
bio4	Temperature Seasonality (standard deviation *100)	7.2	88
tmax12	Maximum Temperature of December	6	94
tmean3	Mean Temperature of March	2.5	96.5
tmin1	Minimum Temperature of January	1.2	97.7
alt	Elevation	1	98.7
tmean9	Mean Temperature of September	0.9	99.6
tmean1	Mean Temperature of January	0.3	99.9

To eliminate the influence of collinearity on the modeling process and results interpretation, a strong correlation factor with a correlation coefficient higher than 0.8 was eliminated. Pearson correlation analysis was carried out on the twelve main contribution factors in Table 1, and the results are shown in Table 2. The correlation coefficients of the twelve variables in Table 2 are less than 0.8. The twelve variables were selected as the dominant environmental variables affecting the distribution of *Z. bungeanum*. The MaxEnt model was reconstructed based on the selected dominant environmental variables.

**Table 2:** Pearson correlation coefficient of dominant environmental variables

Code	alt	prec8	tmax12	bio4	tmax11	bio15	tmean3	tmax3	tmax2	tmin1	tmean9
prec8	0.064^b^										
tmax12	0.723^a^	0.400^b^									
bio4	0.341^b^	− 0.504^b^	− 0.611^b^								
tmax11	0.281^a^	0.302^b^	0.709^b^	− 0.677^b^							
bio15	0.003^b^	0.021^b^	− 0.107^b^	0.110^b^	− 0.045^b^						
tmean3	0.041^a^	0.120^b^	0.601^b^	− 0.584^b^	0.657^b^	− 0.052^b^					
tmax3	0.651^a^	0.122^b^	0.572^b^	− 0.560^b^	0.629^b^	− 0.051^b^	0.552^b^				
tmax2	0.703^b^	0.259^b^	0.736^b^	− 0.717^b^	0.747^b^	− 0.088^b^	0.709^b^	0.636^b^			
tmin1	0.258^b^	0.342^b^	0.774^b^	− 0.734^b^	0.714^b^	− 0.107^b^	0.609^b^	0.577^b^	0.690^b^		
tmean9	0.534^b^	− 0.020^b^	0.380^b^	− 0.240^b^	0.307^b^	− 0.008^a^	0.262^b^	0.406^b^	0.311^b^	0.389^b^	
tmean1	0.410^b^	0.385^b^	0.768^b^	0.769^b^	0.703^b^	− 0.066^b^	0.603^b^	0.596^b^	0.691^b^	0.776^b^	0.381^b^

^a^Means the difference is significant at the 0.05 level; ^b^Means the difference is extremely significant at the 0.01 level

### Model optimization and validation

The settings of regularization multiplier (RM) and feature classes (FC) in the Maxent algorithm are used to balance model fitting and complexity, and determine the types of constraints allowed in the model [13]. Akaike information criterion (AIC) quantity reflects the fitting and complexity of the model, which is an excellent standard to measure the performance of the model. A model with a minimum AICc value (i.e., delta AICc = 0) is considered the best model [14]. The area under the ROC curve (AUC), true skill statistic (TSS) and Cohen’s Kappa (Kappa) were used to evaluate model accuracy [15].

In the mode of default setting (RM = 1.0, FC = LQHPT), the delta AICc was 206.7, AUC_DIFF_ was 0.052 and TSS was 0.521 (Table 3). The goodness of model fitting is not enough, and the accuracy is not very high. Under optimized settings (RM = 2.5, FC = LQHP), the delta AIC value was the lowest, the AUC_DIFF_ value (difference between the training AUC value and the test AUC value) reduced to 0.031, and the value of mean AUC, mean TSS, mean Kappa increased to 0.989, 0.803, 0.789, respectively. The degree of over fitting and complexity of the optimized model were reduced and model performed “excellent” after optimization.

**Table 3:** Model performance under default and optimized settings

	Default	Optimization
RM	1.0	2.5
FC	LQHPT	LQHP
Mean AUC	0.965	0.989
AUC_DIFF_	0.052	0.031
Mean TSS	0.521	0.803
Mean Kappa	0.752	0.789
delta AICc	206.7	0

*RM* regularization multiplier, *FC* feature combination, *AUC* area under the ROC curve, *AUC*_*DIFF*_ the difference between the training AUC and the test AUC, *TSS* true skill statistic, *AIC* Akaike information criterion

### Potential suitable distribution areas

**Fig. 1:**
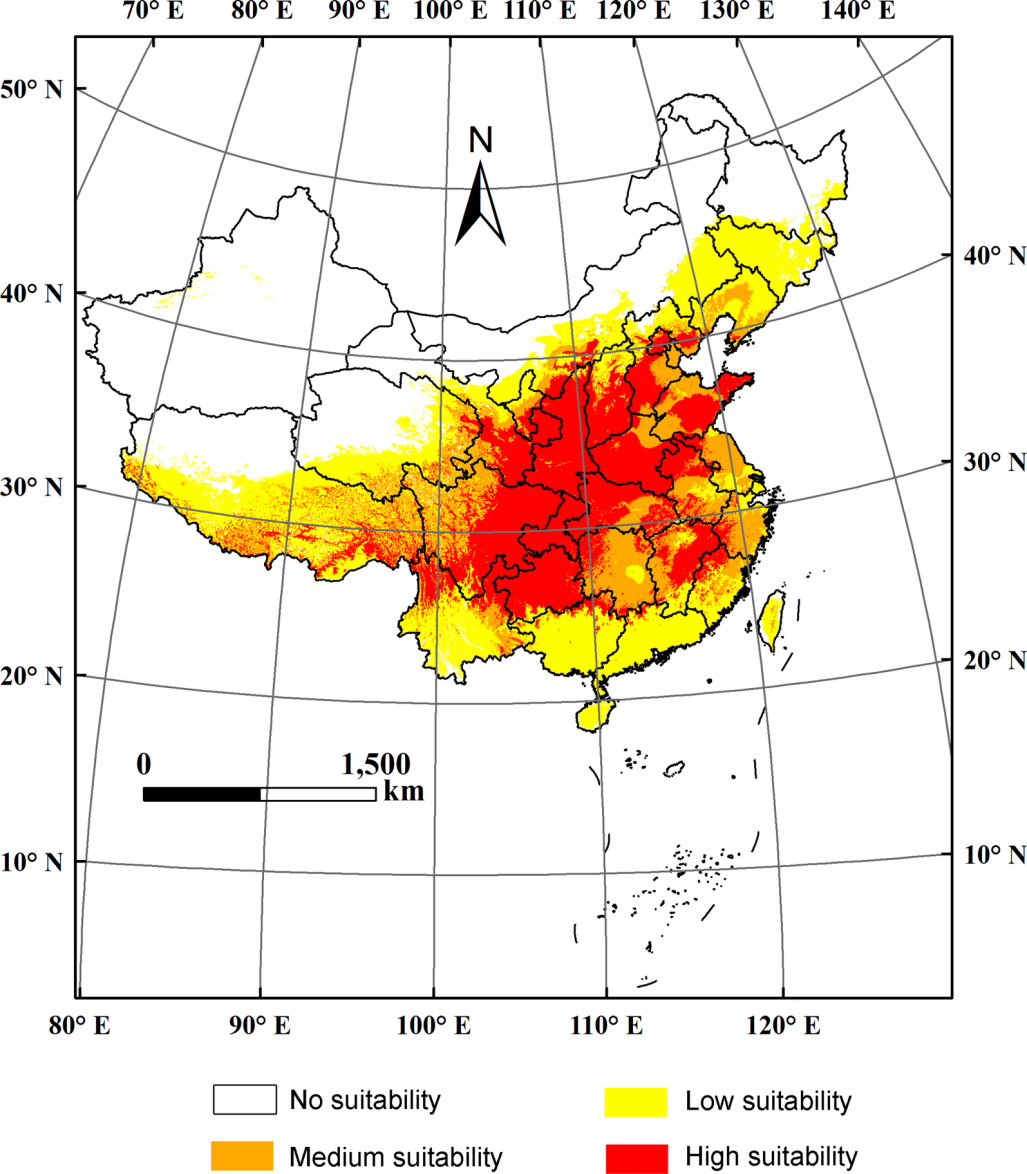
Potential distribution of *Zanthoxylum bungeanum* Maxim. in China (modified from Yuan et al. [16]. Written permission was obtained with license number of 4881970412036)

The potential suitable distribution regions are shown in Fig. 1 (map source: modified from Yuan et al. [16]) and the predicted areas in different provinces are listed in Table 4. The potential area suitable for distribution was divided into four grades. *Z. bungeanum* is distributed in the subtropical and mid-eastern warm temperate regions. It is located in the east of the Qinghai-Tibet Plateau, mainly in the area of the eastern part of the Yunnan-Guizhou Plateau, Qinling Mountains, Daba Mountains, Taihang Mountains and Dabie Mountains. The high suitable areas are mainly in the Yangtze River and Yellow River basins. The total area of suitable habitat (high and medium suitability) is 3.05 × 10^6^ km^2^, occupying 32% of China’s total land area. The area of high suitability (1.93 × 10^6^ km^2^) is larger than that of medium suitability (1.13 × 10^6^ km^2^). The provinces with large areas of high suitability are Sichuan, Shaanxi, Guizhou, Henan, Hubei and Gansu.

**Table 4:** Predicted area suitable for distribution of *Zanthoxylum bungeanum* Maxim

Province	No suitability		Low suitability		Medium suitability		High suitability	
	Predicted area (10^4^ km^2^)	Area ratio (%)^a^	Predicted area (10^4^ km^2^)	Area ratio (%)^a^	Predicted area (10^4^ km^2^)	Area ratio (%)^a^	Predicted area (10^4^ km^2^)	Area ratio (%)^a^
Sichuan	0.33	0.72	6.59	14.48	11.04	24.24	27.58	60.57
Shaanxi	0.00	0.00	0.29	1.41	0.39	1.93	19.70	96.68
Guizhou	0.00	0.00	0.70	4.42	0.47	2.94	14.79	92.66
Henan	0.00	0.00	0.00	0.00	2.66	16.47	13.47	83.52
Hubei	0.00	0.00	0.18	1.01	3.93	22.40	13.45	76.59
Gansu	19.89	47.89	5.07	12.21	4.43	10.67	12.14	29.23
Tibet	39.57	34.61	50.08	43.80	12.83	11.22	11.85	10.36
Shanxi	0.07	0.46	4.28	26.82	0.98	6.16	10.62	66.54
Yunnan	1.64	4.79	17.93	52.31	5.38	15.69	9.33	27.21
Shandong	0.01	0.06	0.18	1.16	7.00	45.43	8.22	53.35
Chongqing	0.00	0.00	0.00	0.00	0.04	0.54	7.69	99.41
Hebei	1.63	8.31	5.29	26.94	5.06	25.77	7.65	38.97
Jiangxi	0.01	0.07	2.81	18.38	4.84	31.66	7.62	49.89
Anhui	0.00	0.00	1.45	10.86	5.57	41.69	6.34	47.47
Hunan	0.00	0.00	1.11	5.74	12.86	66.34	5.41	27.92
Inner Mongolia	91.89	71.17	31.17	24.14	2.80	2.17	3.26	2.52
Fujian	0.01	0.08	5.45	49.57	3.16	28.72	2.38	21.66
Guangxi	0.00	0.00	17.42	83.10	1.24	5.91	2.30	10.97
Qinghai	40.62	56.93	21.34	29.92	7.60	10.65	1.79	2.50
Ningxia	0.05	1.02	2.06	39.17	1.54	29.25	1.61	30.51
Jiangsu	0.06	0.57	2.26	23.22	5.84	59.86	1.60	16.38
Zhejiang	0.03	0.33	2.18	23.05	5.86	61.92	1.39	14.68
Beijing	0.00	0.00	0.26	14.94	0.15	8.48	1.32	76.71
Guangdong	0.04	0.26	14.72	94.27	0.30	1.90	0.56	3.57
Liaoning	0.01	0.06	10.06	64.19	5.28	33.69	0.32	2.06
Tianjin	0.00	0.00	0.00	0.28	1.11	91.22	0.11	8.68
Taiwan	0.97	30.37	2.06	64.60	0.14	4.41	0.02	0.60
Shanghai	0.00	0.00	0.56	94.75	0.03	4.41	0.00	0.29
Heilongjiang	49.05	90.10	5.39	9.91	0.00	0.00	0.00	0.00
Xinjiang	174.92	99.60	0.70	0.40	0.00	0.00	0.00	0.00
Jilin	2.28	10.72	18.95	89.02	0.06	0.27	0.00	0.00
Hong Kong	0.00	0.00	0.09	100.31	0.00	0.00	0.00	0.00
Hainan	0.73	25.18	2.18	74.99	0.00	0.00	0.00	0.00
Total area	423.88		232.90		112.56		192.50	

^a^Refers to the ratio of predicted area to the corresponding province’s total land area

### Relationship between environmental variables and geographical distribution

**Fig. 2:**
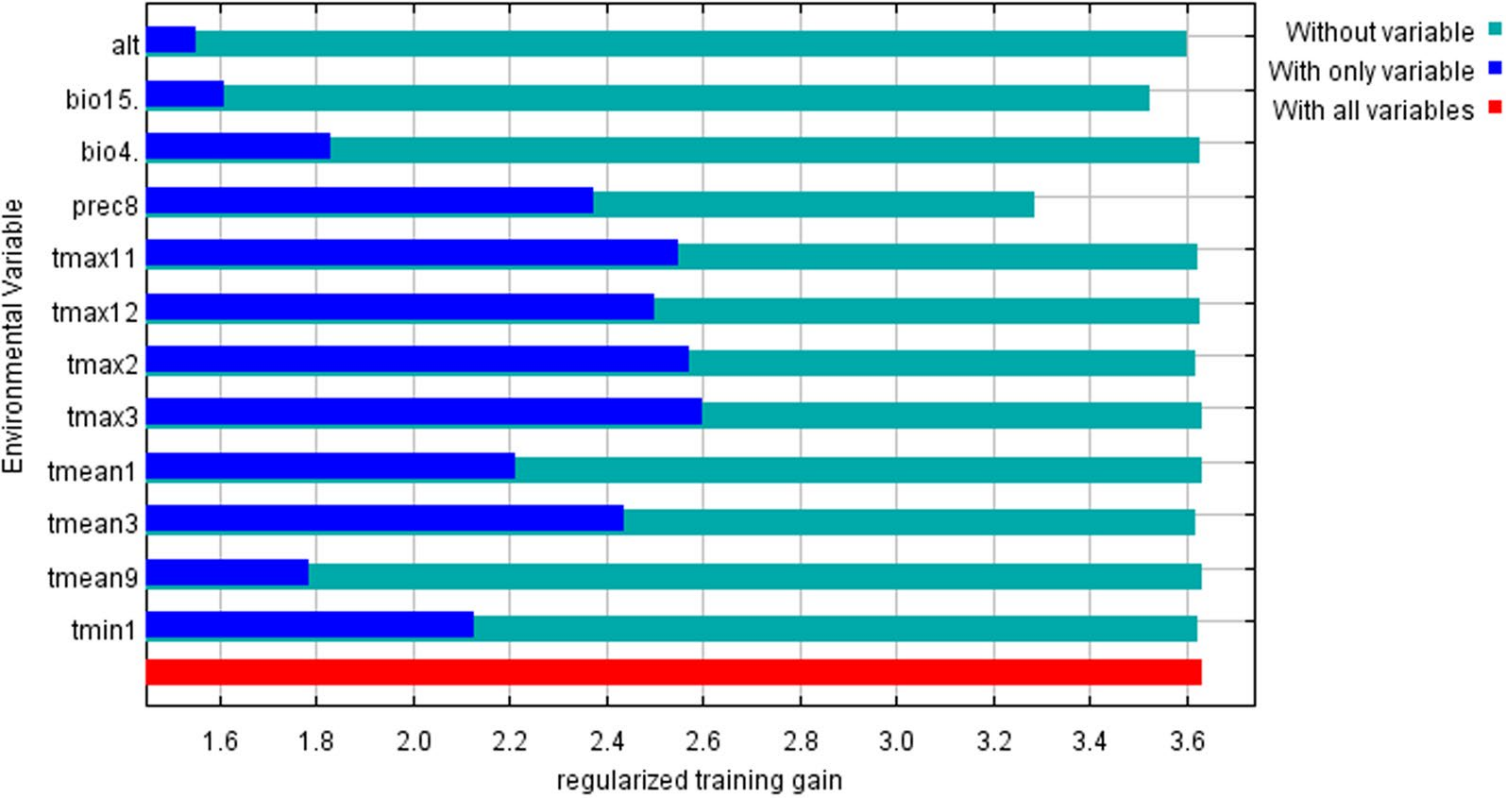
Importance of environmental variables to *Zanthoxylum bungeanum* Maxim. by Jackknife test

The Jackknife test (Fig. 2) showed that the distribution of *Z. bungeanum* was mainly restricted by temperature. Maximum temperature of March (tmax3), February (tmax2), November (tmax11), December (tmax12), and mean temperature of March (tmean3) are the key environmental variables affecting distribution. The training gains are all above 2.4.

**Fig. 3:**
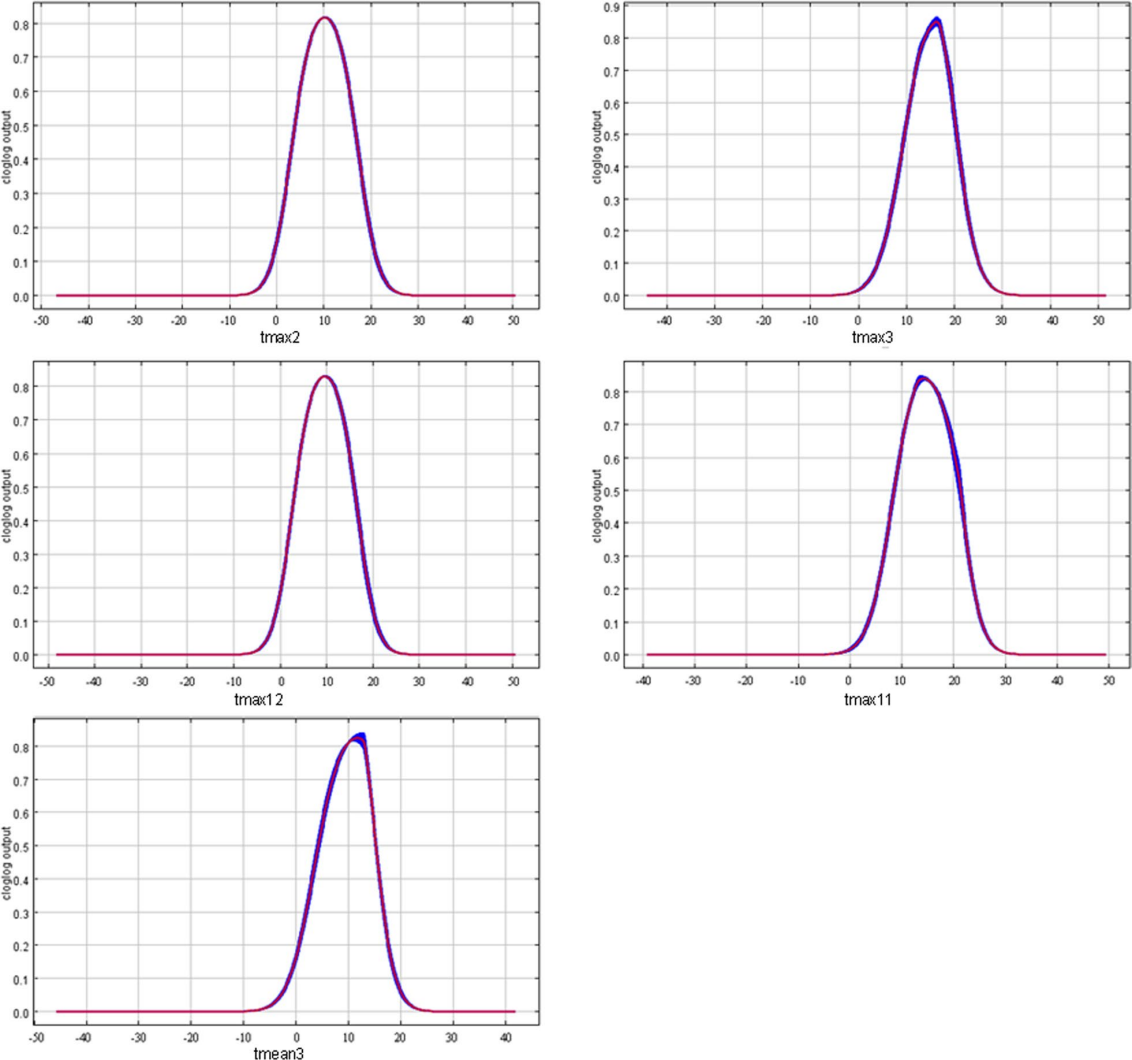
Response curves of environmental variables to distribution probability

According to the response curves of key environmental variables, the response intervals of each factor are obtained as shown in Fig. 3. Based on the probability distribution logic output value of 0.4, the range (and optimum value) of the key environmental variables limiting the distribution of *Z. bungeanum* are the maximum temperature in February of 2.8–17.7 °C (10.4 °C), the maximum temperature in March of 8.6–21.4 °C (16.3 °C), the maximum temperature in December of 2.5–17.1 °C (9.9 °C), the maximum temperature in November of 7.7–22.2 °C (14.5 °C) and the mean temperature in March of 3.2–16.2 °C (12.0 °C). The distribution probability rises with the increase of the value of each key environmental variable before optimum value and drops after optimum value.

## Discussion

In this work, the MaxEnt model was used to model the potential distribution of *Z. bungeanum* in China based on the selected dominant environmental variables. The model accuracy was high (AUC = 0.989, TSS = 0.803, Kappa = 0.789). The high and medium suitability areas are similar to the actual main production areas of *Z. bungeanum* in China. The veracity of the model is influenced not only by types of environmental factors but also by the amount of species distribution points [17]. The result of effects of sample size on accuracy of species distribution models reported by Stockwell and Peterson [18] shows that the average success rate of coarse surrogate model and machine-learning methods is 90% of maximum at ten sample points and reaches maximum accuracy at 100 sample points. The number of sample points used to construct the model reached 127 in this work, which may be the reason for the high accuracy of the simulation results. However, the equilibrium degree of the distribution of samples, and the spatial scale and limitations of the model itself will bring some uncertainties to the modeling results [5, 19–21], which need further study and improvement in the future.

According to the results of the MaxEnt model, the distribution of *Z. bungeanum* is mainly in the subtropical and mid-eastern warm temperate regions, which is consistent with the report in *Flora Reipublicae Popularis Sinicae* [22]. In the subtropical climate region, the solar elevation angle is large and the temperature is high in summer. Southern monsoons bring abundant precipitation, and the rain and heat occur in the same period. The warm temperate zone in the central and eastern part of the country is characterized by hot and rainy summers, cold and dry winters, and distinct seasons. These climatic conditions may be an important factor limiting the distribution of *Z. bungeanum*. This species also has a certain suitable range in the western plateau climate areas of western Sichuan, eastern Tibet and eastern Qinghai. The plateau climate is characterized by strong solar radiation, significant diurnal temperature difference, low temperature, high wind and harsh climate [23], which indicates that *Z. bungeanum* has relatively strong adaptability. The Qinling Mountains-Huaihe River line is the boundary between temperate monsoon climate and subtropical monsoon climate in China, as well as between semihumid and humid regions. *Z. bungeanum* has high adaptability in this area, with high quality and yield represented by places such as Wudu and Qinan in Gansu Province, Hancheng and Fengxian in Shaanxi Province, Laiwu in Shandong Province, Ruicheng in Shanxi Province and Shexian in Hebei Province. This further illustrates that *Z. bungeanum* can adapt to a variety of ecological environments. Western Sichuan and Guizhou provinces in southwest China present a warm and humid climate, small daily temperature difference, abundant rainfall, and warmth in winter and heat in summer, which provides an appropriate ecological environment for plant growth [24]. There are places in this area with a long cultivation history and high quality of *Z. bungeanum*, such as Hanyuan, Maoxian and Mianning in Sichuan Province, and Zunyi and Bijie in Guizhou Province. The model predicted results are consistent with the actual growth range.

The results of the MaxEnt model showed that the distribution of *Z. bungeanum* was mainly restricted by temperature, especially the maximum temperature of February, March, December, and November and the mean temperature of March. The period of February to March is the germination stage of *Z. bungeanum*. When the average temperature is above 6 °C in spring, buds begin to germinate; when above 10 °C, new shoots begin to grow [25]. The average maximum temperature in February and March is too low, which may easily cause flower organs to be frozen and the fruit to be insufficiently developed. If the temperature is too high, it may lead to premature development, excessive growth of new branches, unbalanced nutrition and underdevelopment of fruits. Therefore, the maximum temperatures of 10.4 °C in February and 16.3 °C in March are the optimum temperatures for the full development of *Z. bungeanum*. The *Z. bungeanum* is not tolerant to severe cold [26]. November to December is the winter season in China. At this time, the temperature directly determines whether *Z. bungeanum* can safely pass through the dormancy period and whether freezing damage occurs [27], which influences the quality and yield to a certain extent. Thus, the maximum temperatures of 9.9 °C in December and 14.5 °C in November are the optimum values for growth.

The species distribution under the ideal state is almost impossible in reality, so it may occur that the predicted area is larger than the actual distribution area. On the other hand, due to the self-adaptability of plants as well as the influence of human activity, plants can survive in areas beyond the original basic niche [17, 28]. In this situation, the modeled species distribution area may be smaller than the actual distribution area. As a horticultural plant affected by human activity, such as irrigation, variety improvement, cultivation management, and market demand, it is possible to expand the distribution area of *Z. bungeanum*, resulting in the predicted distribution area being smaller than the actual. The adoption of more key ecological factors restricting species distribution will undoubtedly improve the accuracy of model simulation. In this work, only the effects of 70 environmental variables on the distribution are considered. The effects of interspecies interaction and human activity are not considered, which may have a certain negative impact on the accuracy of prediction results. It is impossible to consider all environmental factors in a particular model analysis, so it may be more realistic to regard the model as an ideal distribution model [29]. Since data related to impact factors such as artificial introduction, cultivation management, and market demand are difficult to obtain, how to incorporate these factors into the model is a matter that needs to be taken into account in the future.

## Conclusions

The suitable habitat for *Z. bungeanum* were predicted successfully by the MaxEnt based on known distribution data and environmental variables in China. Suitable areas for *Z. bungeanum* to introduction and cultivation were mainly distributed in subtropical and mid-eastern warm temperate regions with a total suitable area of 3.05 × 10^6^ km^2^. The maximum temperature of February, March, December, and November and the mean temperature of March are the key environmental variables limiting the distribution. Only climate and topographical variables were considered for modeling in this work. More environmental variables such as human activity, soil type, vegetation types and interspecies interaction should be concerned in the future to improve the accuracy and precision of model prediction.

## Methods

### Species occurrence data

**Fig. 4:**
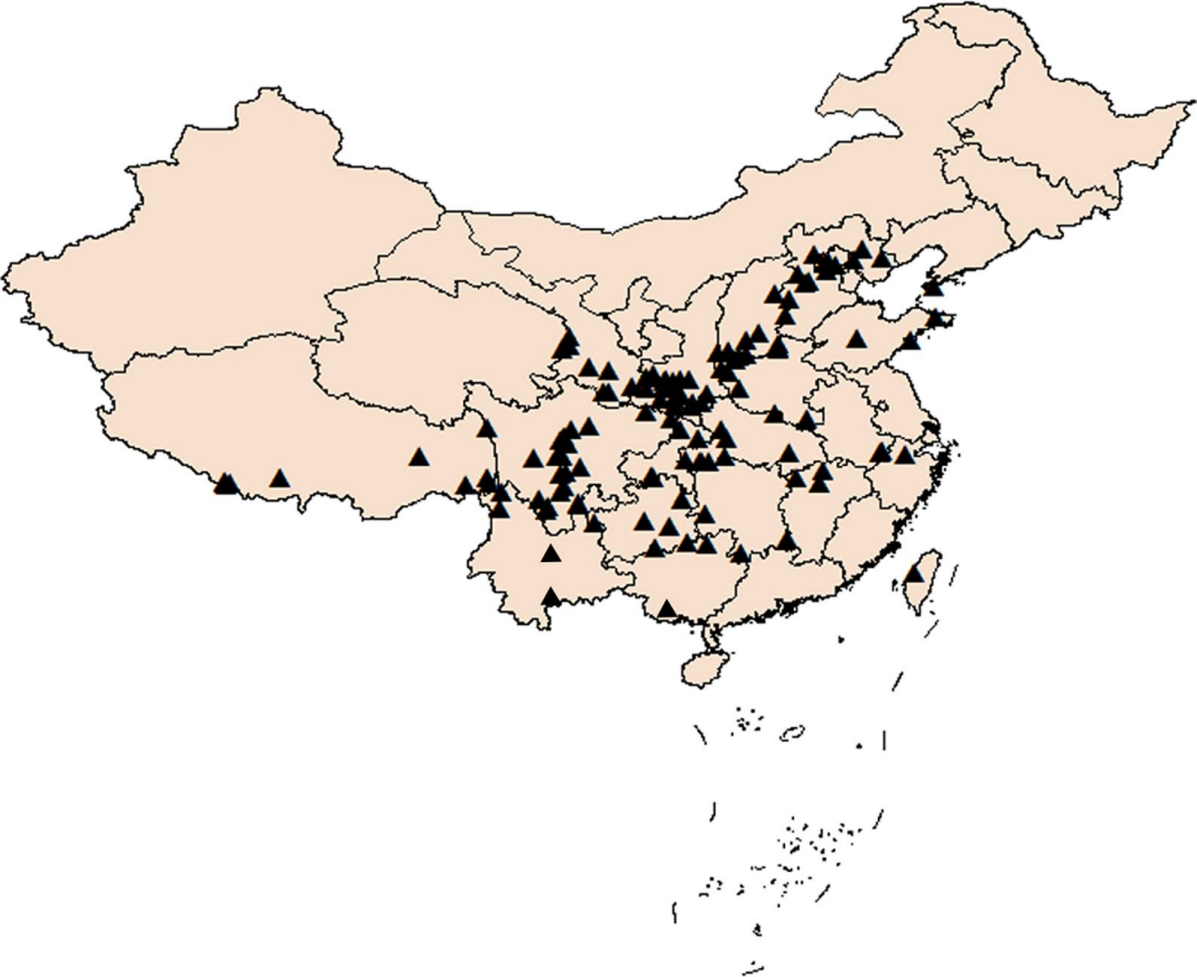
Species occurrence records (modified from Yuan et al. [16]. Written permission was obtained with license number of 4881970412036). Triangle symbol represents natural distribution *Zanthoxylum bungeanum* Maxim. in China

The natural distribution data of *Z. bungeanum* was derived from the sample records of the Global Biodiversity Information Facility (GBIF, https://www.gbif.org/), the Chinese Virtual Herbarium (CVH, http://www.cvh.ac.cn/) and field investigations. The distribution sites with insufficient accuracy and repetition were eliminated. It’s likely that samples near roads and towns would be heavily sampled which cause sampling bias [30]. In this work, the sampling bias was corrected according to the attribute of environment variable. Specifically, in the same cell grid, only one distribution point closest to the center point was reserved. A total of 127 effective sites were obtained in China (Fig. 4, map source: modified from Yuan et al. [16]). The input files in CSV format were generated according to the requirements of the software MaxEnt 3.3.3 k (http://www.cs.princeton.edu/schapire/Maxent/) [31].

### Environmental variables

*Zanthoxylum bungeanum* is a kind of shade-intolerant tree species, with the characteristics of preferring warmth, not cold tolerance, and poor water tolerance of root system [22]. Its growth process is mainly influenced by temperature, precipitation, sunshine, and topography. In this work, a total of 70 environmental variables including 19 bioclimatic variables (bio1-bio19), 48 monthly climatic variables, and three topographical factors were selected based on the biological characteristics of *Z. bungeanum.* The monthly climatic variables were minimum temperature (tmin), maximum temperature (tmax), mean temperature (tmean) and precipitation (prec) of each month. The topographical factors were elevation (alt), slope (slo) and aspect (asp). The environmental variables were list in Additional file 1: Table S1. Climate variables data were derived from WorldClim (http://www.worldclim.org) with the year span of 1970 to 2000. The data set had a spatial resolution of 30 s (~ 1 km^2^). The digital elevation model (DEM) was obtained from Shuttle Radar Topographic Mission (SRTM) (http://srtm.usgs.gov/index.php) and the information of elevation, slope, and aspect were extracted from DEM by ArcGIS [32].

To eliminate the adverse effects of multicollinearity of environmental factors on modeling, the following two steps were conducted [31, 33]. Firstly, the initial environmental variables and species distribution data were imported into MaxEnt to calculate the contribution rate of each environmental variable by jackknife test. The variables with small contribution rate were removed. Then, the Pearson correlation coefficient (*r*) between the remaining environmental variables was calculated by SPSS. The variables with *r* < 0.8 were retained. For the variables with *r* ≥ 0.8, the importance was measured according to their biological significance and contribution rate. After these two processes, twelve variables were obtained for modeling (Table 2).

### Establishment, optimization and evaluation of model

MaxEnt 3.3.3 k software was used for modeling the potential distribution of *Z. bungeanum.* Repeat the operation for 10 times, and cross validation was selected to extract test samples. The contribution rate of environmental variables to the distribution of *Z. bungeanum* was quantitatively studied by the Jackknife method. RM and FC were optimized by calling *ENMevaluate* from *ENMeval* R package (http://www.R-project. org) to avoid overfitted models and improve the accuracy [34, 35]. The model was built with RM changing from 0.5 to 4.0 (increments of 0.5) and several FC combinations (L, LQ, H, LQH, LQHP, LQHPT; where L = linear, Q = quadratic, H = hinge, P = product and T = threshold). *Enmeval* was used to test the above 48 parameter combinations. AIC was used as a criterion to select the best model. The receiver operating characteristic (ROC) curve was used to evaluate and verify the accuracy of the model operation results. The value of area (0–1) under the ROC curve (AUC) can well reflect the accuracy of model prediction. Thus, the model was optimized according to the AIC values (delta AIC) and the difference between the training AUC value and the test AUC value (AUC_DIFF_) [14, 36].

The accuracy of model simulation results is proportional to AUC value. AUC evaluation criteria were divided into five cases: failed (0.50–0.60), poor (0.60–0.70), fair (0.70–0.80), good (0.80–0.90), and excellent (0.90–1.00) [37]. Besides, TSS and Kappa were also selected to evaluate the accuracy because of their characteristic of being not affected by the size of the validation set [15]. The value of Kappa higher than 0.75 means the model performs excellent. TSS is the difference between omission and commission errors [38]. The range of TSS is from -1 to 1. Value of TSS closes to 1 means high accuracy, and value closes to -1 means low accuracy. TSS = 0 means the model is unable to differentiate between omission and commission errors.

The distribution map of *Z. bungeanum* in China was then extracted by spatial analysis technology in ArcGIS. The criteria for classification of habitat suitability according to existence probability were as follows: high suitability (0.6–1), medium suitability (0.4–0.6), low suitability (0.2–0.4) and no suitability (0–0.2) [39].

## Supplementary information

**Additional file 1: Table S1.** List of environmental variables used in model development.

## Data Availability

All data generated or analyzed during this study are included in this published article and its supplementary information files.

## Abbreviations

MaxEnt: The maximum entropy
ROC: Receiver operating characteristic curve
AUC: Area under the ROC curve
RM: Regularization multiplier
FC: Feature classes
AIC: Akaike information criterion
TSS: True skill statistic
Kappa: Cohen’s Kappa
