# Supplementary material for: Predicting distribution of Zanthoxylum bungeanum Maxim. in China
Source: BMC Ecol. 2020 Aug 11;20:46. doi: 10.1186/s12898-020-00314-6 (PMC7422582; doi:10.1186/s12898-020-00314-6)
Supplement: Supplementary file 1 — Additional file 1: Table S1. List of environmental variables used in model development. [file 12898_2020_314_MOESM1_ESM.docx]

**Additional file**

**Table S1** List of environmental variables used in the model development

| Code | Environmental variables | Unit |
| --- | --- | --- |
| bio1 | Annual Mean Temperature | ℃ |
| bio2 | Mean Diurnal Range (Mean of monthly (max temp - min temp)) | ℃ |
| bio3 | Isothermality (bio 2/ bio 7) (* 100) | % |
| bio4 | Temperature Seasonality (standard deviation *100) | ℃ |
| bio5 | Max Temperature of Warmest Month | ℃ |
| bio6 | Min Temperature of Coldest Month | ℃ |
| bio7 | Temperature Annual Range (bio5-bio6) | ℃ |
| bio8 | Mean Temperature of Wettest Quarter | ℃ |
| bio9 | Mean Temperature of Driest Quarter | ℃ |
| bio10 | Mean Temperature of Warmest Quarter | ℃ |
| bio11 | Mean Temperature of Coldest Quarter | ℃ |
| bio12 | Annual Precipitation | mm |
| bio13 | Precipitation of Wettest Month | mm |
| bio14 | Precipitation of Driest Month | mm |
| bio15 | Precipitation Seasonality (Coefficient of Variation) | 1 |
| bio16 | Precipitation of Wettest Quarter | mm |
| bio17 | Precipitation of Driest Quarter | mm |
| bio18 | Precipitation of Warmest Quarter | mm |
| bio19 | Precipitation of Coldest Quarter | mm |
| tmin | Minimum Temperature of Each Month | ℃ |
| tmax | Maximum Temperature of Each Month | ℃ |
| tmean | Mean Temperature of Each Month | ℃ |
| prec | Precipitation of Each Month | mm |
| alt | Elevation | m |
| slo | Slope | ° |
| asp | Aspect | ° |
